# Loss of Activating EGFR Mutant Gene Contributes to Acquired Resistance to EGFR Tyrosine Kinase Inhibitors in Lung Cancer Cells

**DOI:** 10.1371/journal.pone.0041017

**Published:** 2012-07-17

**Authors:** Keisuke Tabara, Rina Kanda, Kahori Sonoda, Takuya Kubo, Yuichi Murakami, Akihiko Kawahara, Koichi Azuma, Hideyuki Abe, Masayoshi Kage, Aki Yoshinaga, Tomoko Tahira, Kenshi Hayashi, Tokuzo Arao, Kazuto Nishio, Rafael Rosell, Michihiko Kuwano, Mayumi Ono

**Affiliations:** 1 Department of Pharmaceutical Oncology, Graduate School of Pharmaceutical Sciences, Kyushu University, Fukuoka, Japan; 2 St. Mary’s Hospital, Kurume, Japan; 3 Department of Diagnostic Pathology, Kurume University Hospital, Kurume, Japan; 4 Division of Respirology, Neurology and Rheumatology, Department of Internal Medicine, Kurume University School of Medicine, Kurume, Japan; 5 Division of Genome Analysis, Research Center for Genetic Information, Medical Institute of Bioregulation, Kyushu University, Fukuoka, Japan; 6 Department of Genome Biology, Faculty of Medicine, Kinki University, Osaka, Japan; 7 Catalan Institute of Oncology, Hospital Germans Trias i Pujol, Badalona, Spain; 8 Pangaea Biotech, S.L., USP Dexeus University Institute Barcelona, Barcelona, Spain; 9 Laboratory of Molecular Cancer Biology, Department of Clinical Pharmaceutics, Graduate School of Pharmaceutical Sciences, Kyushu University, Fukuoka, Japan; University of Colorado, United States of America

## Abstract

Non-small-cell lung cancer harboring epidermal growth factor receptor (EGFR) mutations attains a meaningful response to EGFR-tyrosine kinase inhibitors (TKIs). However, acquired resistance to EGFR-TKIs could affect long-term outcome in almost all patients. To identify the potential mechanisms of resistance, we established cell lines resistant to EGFR-TKIs from the human lung cancer cell lines PC9 and11–18, which harbored activating EGFR mutations. One erlotinib-resistant cell line from PC9 and two erlotinib-resistant cell lines and two gefitinib-resistant cell lines from 11–18 were independently established. Almost complete loss of mutant delE746-A750 EGFR gene was observed in the erlotinib-resistant cells isolated from PC9, and partial loss of the mutant L858R EGFR gene copy was specifically observed in the erlotinib- and gefitinib-resistant cells from 11–18. However, constitutive activation of EGFR downstream signaling, PI3K/Akt, was observed even after loss of the mutated EGFR gene in all resistant cell lines even in the presence of the drug. In the erlotinib-resistant cells from PC9, constitutive PI3K/Akt activation was effectively inhibited by lapatinib (a dual TKI of EGFR and HER2) or BIBW2992 (pan-TKI of EGFR family proteins). Furthermore, erlotinib with either HER2 or HER3 knockdown by their cognate siRNAs also inhibited PI3K/Akt activation. Transfection of activating mutant EGFR complementary DNA restored drug sensitivity in the erlotinib-resistant cell line. Our study indicates that loss of addiction to mutant EGFR resulted in gain of addiction to both HER2/HER3 and PI3K/Akt signaling to acquire EGFR-TKI resistance.

## Introduction

Non-small-cell lung cancer (NSCLC) is one of the most widespread malignant cancers and a leading cause of death worldwide. Development of anticancer drugs that target epidermal growth factor receptor (EGFR) has improved treatment of NSCLC. Two representative EGFR-tyrosine kinase inhibitors (EGFR-TKIs), gefitinib and erlotinib, have a common quinazoline structure and have been approved for the treatment of progressive NSCLC. Both erlotinib and gefitinib show similar kinase inhibition selectivity based on quantitative analysis of small molecule-kinase interaction maps for 38 kinase inhibitors [1], and show therapeutic efficacy against progressive NSCLC patients [2]–[4].

The most common activating EGFR mutations are in-frame deletion in exon 19 (delE746-A750) and the point mutation replacing leucine with arginine at codon 858 of exon21 (L858R) [5]–[9]. These two major mutations account for 85–90% of all mutations and enhance the therapeutic efficacy of EGFR-targeted drugs [10]–[13]. Furthermore, these activating mutations gained addiction to EGFR in lung cancer cells, resulting in enhanced susceptibility to EGFR-TKI such as gefitinib and erlotinib [6], [14]–[16].

One serious problem with EGFR-TKI treatment is the appearance of drug-resistant tumors. For acquired resistance, secondary mutation in the EGFR gene T790M [16]–[18] or alternative EGFR-independent activation of cell growth signaling pathways including c-Met activation is well-known [19], [20]. The loss of PTEN expression is one of the acquired resistant mechanisms, which was demonstrated by isolating gefitinib-resistant mutants from PC9 cells which harbor activating mutation of EGFR [21], [22]. In addition to the well-characterized causes of drug resistance in lung cancer patients, elucidation of further mechanism for acquired resistance is essential for the development of new EGFR-targeted drugs.

In this present study, erlotinib- and gefitinib-resistant cell lines were established from two human lung cancer cell lines, PC9 cells harboring delE746-A750 mutation and 11–18 cells harboring L858R mutation, respectively. Surprisingly, the partial or complete loss of the mutant EGFR gene copy was observed in the erlotinib- and gefitinib-resistant cell lines. The clinical significance of the loss of mutant EGFR is discussed in relation to its close association with acquisition of drug resistance to EGFR-TKIs in NSCLC patients.

**Table 1:** Comparison of sensitivity to various drugs between erlotinib- and gefitinib- resistant cell lines and their drug sensitive parental counterparts, PC9 and 11–18 cells.

		Relative drug resistancea)				
Cell lines	Erlotinib	Gefitinib	Lapatinib	SU11274	BIBW2992	Cisplatin
PC9	1	1	1	1	1	1
PC9/ER1	249	168	4.6	1	1952	1.4
11–18	1	1	1	1	1	1
11–18/ER1-7	24	26	1.1	1.1	1.4	0.3
11–18/ER2-1	110	64	1.6	1.2	2.1	1.7
11–18/GEF10-1	>34	36	5.2	0.8	6.9	ntb)
11–18/GEF20-1	>34	32	5.5	0.9	4.6	nt

a) IC50 values are calulated from logit regression line from triplicate assays. IC50 values (µM) for erlotinib, gefitinib, lapatinib, SU11274, BIBW2992 and cisplatin are 0.04, 0.03, 3.16, 3.97, 0.00021 and 2.07 in PC9, 9.96, 5.04, 14.54, 3.97, 0.41 and 2.89 in PC9/ER1, 0.87, 0.32, 2.57, 4.22, 0.35 and 5.37 in 11–18, 20.88, 8.32, 2.83, 4.64, 0.49 and 1.61 in 11–18/ER1-7, 95.7, 20.48, 4.11, 5.06, 0.74 and 9.13 in 11–18/ER2-1, >29.58, 11.52, 13.36, 3.38 and 2.41 in 11–18/GEF10-1, and >29.58, 10.24, 14.14, 3.80 and 1.61 in 11–18/GEF20-1 respectively. The relative resistance is defined as the IC50 value divided by the IC50 value of the parental PC9 or 11–18.

b) nt, not tested.

## Materials and Methods

### Cell Culture and Reagents

Human lung cancer cell lines, PC9, QG56 and 11–18 were cultured in RPMI medium supplemented with 10% fetal bovine serum (FBS) as described previously [14], [21]. PC9 and QG56 were kindly provided by Dr. Yukito Ichinose (National Hospital Organization Kyushu Cancer Center, Fukuoka, Japan), and 11–18 was by Dr. Kazuhiko Nakagawa (Kinki University, Osaka, Japan). Erlotinib was kindly provided by F. Hoffman-La Roche Ltd, gefitinib was by AstraZeneca Inc. BIBW2992 was purchased from Selleck Chemicals, SU11274 and wortmannin were from Calbiochem, LY294002 was from Cell Signaling Technology and Lapatinib was from Toronto Research Chemicals. Anti-HER2 and anti-phospho-HER2 antibodies were purchased from Upstate Biotechnology, Anti-phospho-EGFR, anti-EGFR, anti-phospho-HER3, anti-phospho-c-Met, anti-phospho-Akt, anti-Akt, anti-PTEN, anti-phospho-ERK1/2, anti-ERK1/2, and mutation-specific (L858R in exon 21 and deletion E746-A750 in exon 19) antibodies were from Cell Signaling Technology, anti-HER3 and anti-c-Met antibodies were from Santa Cruz Biotechnology, anti-α-tubulin antibody was from Sigma-Aldrich, and anti-GAPDH antibody was from Trevigen. Complementary DNAs (cDNA) for EGFR and activating mutant EGFR were kindly provided by Dr. Willam Pao and Dr. Nishio. Cells were transfected with cDNA using Lipofectamine LTX, PLUS reagent and Opti-MEM (Invitrogen) according to the manufacturer’s recommendations. Recombinant human EGF was purchased from PEPROTECH. The small interfering RNAs (siRNA) corresponding to HER2 (5′-AAACGUGUCUGUGUUGUAGGUGACC-3′), HER3 (5′-GGCAGUGUAUAAUCUAUCUCCACUA-3′) and PIK3CA (5′-CCCUAUUGGUGGUGUUACUGGAUCAAAU-3′) were purchased from Invitrogen, and corresponding to EGFR (5′-GAGGAAAUAUGUACUACGA-3′) were purchased from Sigma-Aldrich. Cells were transfected with siRNA duplexes using Lipofectamine RNAiMAX and Opti-MEM (Invitrogen) according to the manufacturer’s recommendations.

### Cytotoxicity Assays

Exponentially growing cell suspensions were seeded into each well and the following day the indicated concentrations of the different drugs were added. After incubation for 72 hr, cytotoxicity was determined as described previously [21].

### Western Blot Analysis

Cells were rinsed with ice-cold PBS and lysed in Triton X-100 buffer (50 mmol/L HEPES, 150 mmol/L NaCl, 50 mmol/L NaF, 1% Triton X-100, and 10% glycerol, containing 5 mmol/L EDTA, 1 mmol/L phenylmethylsulfonyl fluoride, 10 µg/mL aprotinin, 10 µg/mL leupeptin, and 1 mmol/L sodium orthovanadate), and proteins from cell lysates were separated by SDS-PAGE and transferred to Immobilon membranes (Millipore Corp.), as described previously (21). After transfer, the membranes were incubated in blocking solution, probed with the different antibodies, washed, and visualized using horseradish peroxidase-conjugated secondary antibodies (GE Healthcare) and enhanced chemiluminescence reagent (Amersham).

**Figure 1:**
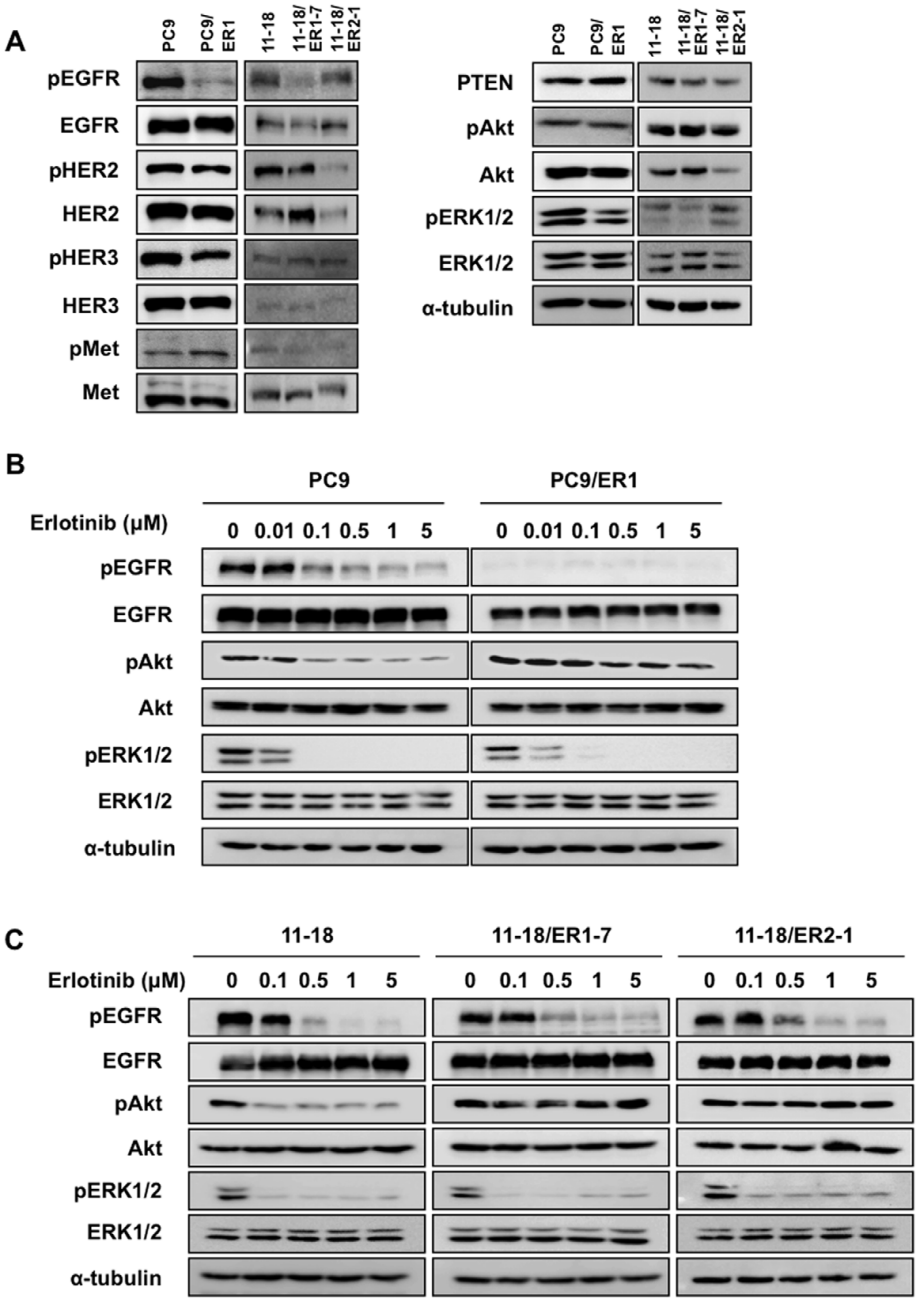
Comparison of protein expression of EGFR family proteins and the down-stream molecules in erlotinib-resistant cell lines in the absence or presence of erlotinib. A, Western blot analysis showing the expression of pEGFR, EGFR, pHER2, HER2, pHER3, HER3, pc-Met, c-Met, PTEN, pAkt, Akt, pERK1/2, and ERK1/2 proteins, and α-tubulin as a loading control. B, Exponentially growing PC9 and PC9/ER1 cells were exposed to various doses of erlotinib for 5 hr, and followed by Western blot analysis. C, Exponentially growing 11–18, 11–18/ER1-7, and 11–18/ER2-1 cells were exposed to various doses of erlotinib for 5 hr, and followed by Western blot analysis.

### Human RTK Arrays

Proteome Profiler human phospho-RTK antibody arrays (R&D Systems) were used according to the manufacturer’s instructions.

### PLACE-SSCP Analysis

PLACE-SSCP analysis was performed as described previously [23]–[25]. Genomic segments containing mutated sequences were amplified by PCR from DNAs extracted from five cell lines (11–18, 11–18/ER1-7, 11–18/ER2-1, 11–18/GEF10-1, and 11–18/GEF20-1), and normal human umbilical vein endothelial cells (HUVECs) which were purchased from Lonza Walkersville Inc. To analyze the L858R mutation, exon 21 of the EGFR gene was amplified using primers (forward: 5′-ATTCAGGGCATGAACTACTTGG-3′,reverse: 5′-GTTACCTCCTTACTTTGCCTC CT-3′) and TaKaRa ExTaq polymerase (Takara BIO Inc.). The obtained trace files (.fsa) served as input files to QSNPlite for analysis [26].

### Allele Quantification

QSNPlite calculates the peak-height ratio (R_h_) of two alleles the (wild-type and mutant allele) in each SSCP run. To estimate the copy number of alleles per cell in each of the five test cells (see previous subsection), mixing experiments were performed using HUVECs as a reference. In this case, HUVECs were presumed to carry two copies of the wild-type allele per cell. Rh values for each of the five test cells were obtained as the median of five replicates, each of which consist of test cells alone and equal-part mixture of the test and the reference. The copy number of the two alleles in the test cells was estimated from the difference of R_h_ values between the tested cells alone and the equal-part mixture, as follows: Suppose the test cells carry X copy per cell of wild-type EGFR, and Y copy per cell of mutant EGFR. Then, the R_h_ of SSCP analysis for test cells, R_h_(test), is represented by; R_h_(test) = M×(X/Y), where M is an allele-dependent constant that comes from the differences in PCR-amplification efficiency, labeling efficiency, and the shape of peak, between wild-type and mutant alleles. Similarly, R_h_ of an equal-part mixture of test cells and the reference, R_h_(mix), is given in the following equation.
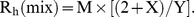



From the two equations above, X and Y are obtained as follows.
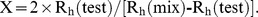


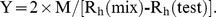



The equations above implicate that absolute copy-number of the mutant allele in the tested cells cannot be estimated, because M is unknown. However, relative values of copy-numbers for the same mutant allele in different test cells can be estimated, because M is a constant.

**Figure 2:**
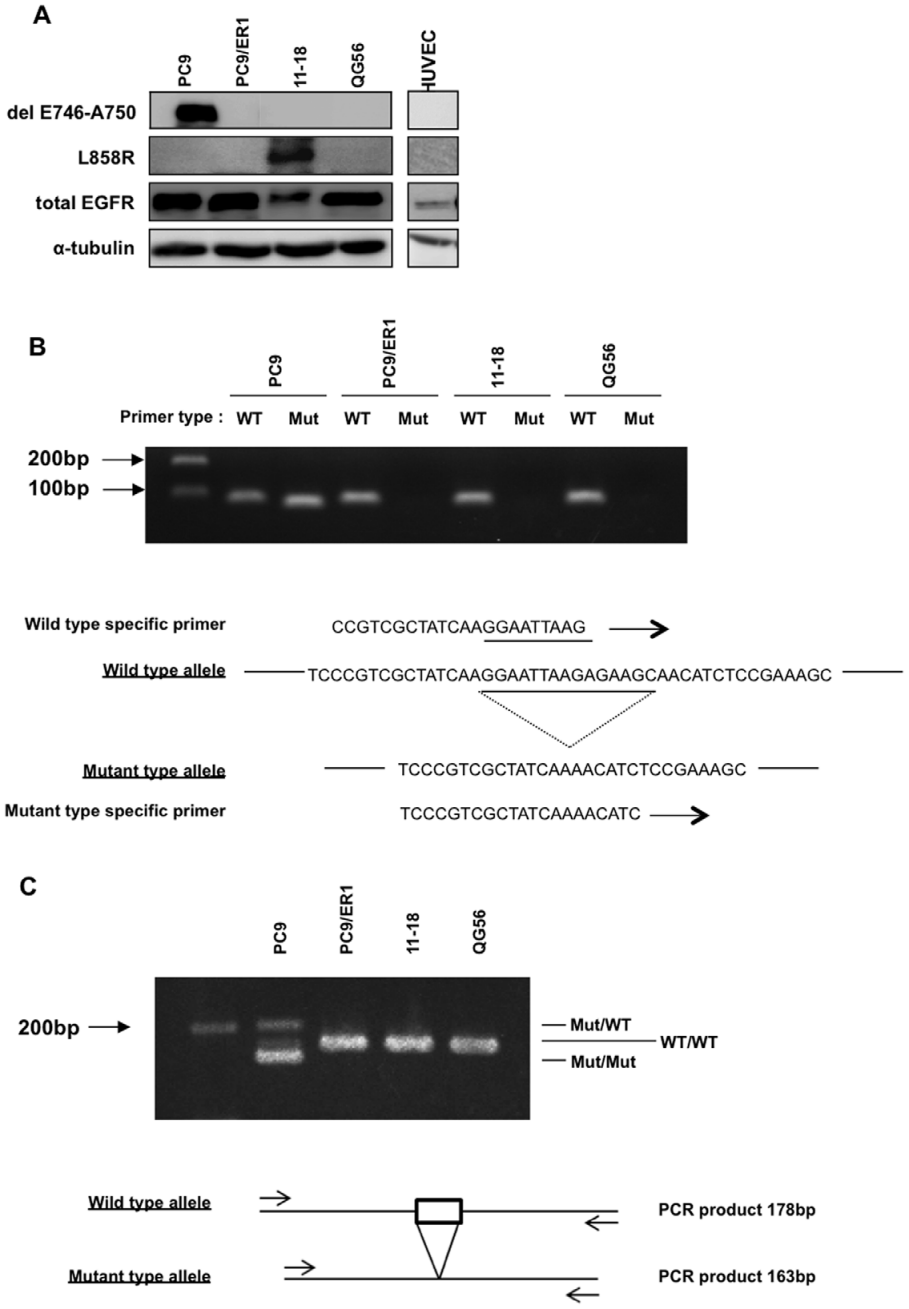
Comparison of EGFR gene and protein expression between PC9 and PC9/ER1 cells. A, Western blots showing expression of delE746-A750 EGFR and total EGFR in PC9 and PC9/ER1 cells. B, Detection of wild-type and mutation sequences using specific primers. PC9 cells show both wild-type and mutant-specific bands, while PC9/ER1, 11–18, and QG56 cells show only the wild-type-specific band. Mut, mutant exon19, and WT, wild-type exon19. C, PCR analysis shows heteroduplex (Mut/WT) and homoduplex (WT/WT and Mut/Mut) in PC9 cells, and only homoduplex (WT/WT) in PC9/ER1 cells, 11–18 cells harboring L858R mutation, and QG56 cells harboring wild-type EGFR.

**Figure 3:**
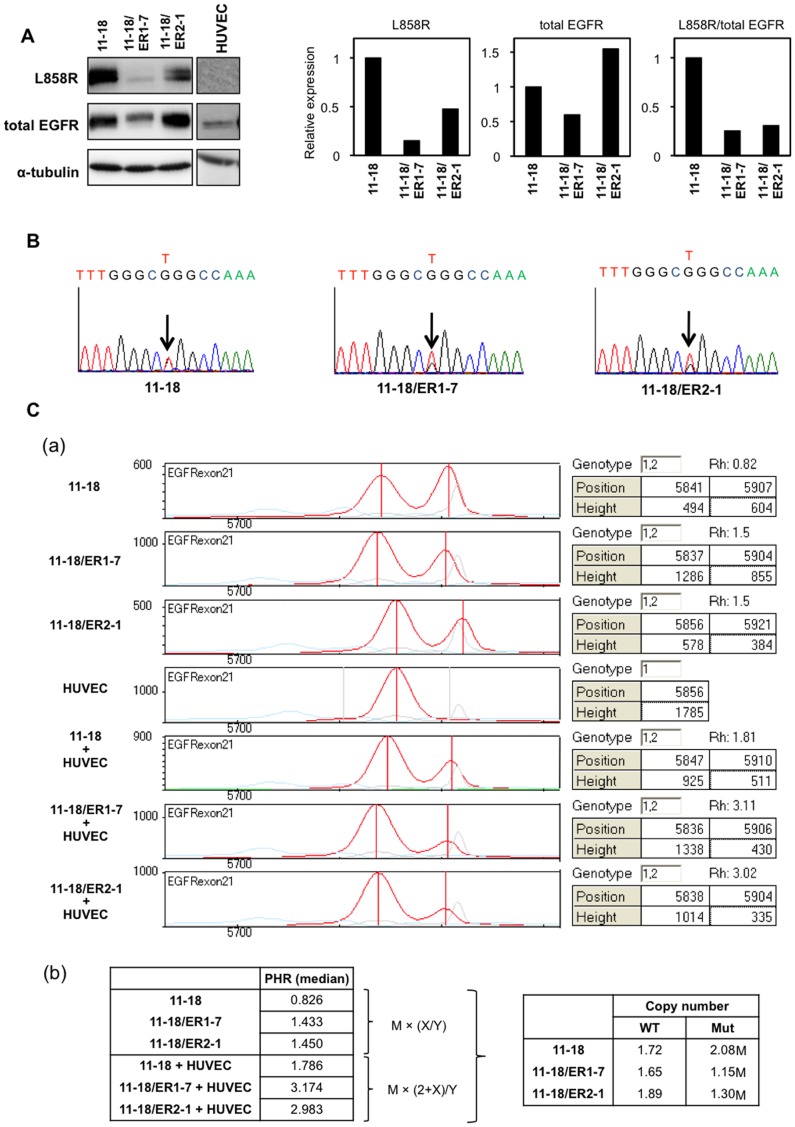
Comparison of EGFR gene and protein expression in 11–18, 11–18/ER1-7, and 11–18/ER2-1. A, Western blot analysis using L858R-specific antibody and total EGFR antibody recognizing both wild-type and mutant EGFR. Expression levels of mutant EGFR (L858R), total EGFR, and L858R versus total EGFR (L858R/total EGFR) are normalized by their expression levels in 11–18 cells. Western blot analysis with primary human endothelial cell (HUVEC) shows expression of total EGFR, but not mutant L858R EGFR. B, DNA sequences reads of 15 bases around the L858R mutation in 11–18, 11–18/ER1-7, and 11–18/ER2-1 cells are compared. Arrows indicate base at 2573. Note that the nucleotide of 11–18 at 2573 was G/T heterozygote. C, PLACE-SSCP analysis of DNA samples of 11–18, 11–18/ER1-7, and 11–18/ER2-1 cells in the absence or presence of equal amounts of DNA from human vascular endothelial cells (HUVECs) are shown. Two peaks show wild-type (WT) and mutant (Mut) EGFR gene (a). Based on the equations shown in Materials and Methods, copy number changes of the wild-type and mutant EGFR genes are estimated (b).

### PCR Analysis

To analyze the deletion mutation, exon 19 of the EGFR gene was amplified using the following PCR forward primers:

wild-type specific, 5′-CCGTCGCTATCAAGGAATTAAG-3′ mutant specific, 5′-TCCCGTCGCTATCAAAACATC-3′ both wild-type and mutant type, 5′-ATGTGGCACCATCTCACAATTGCC-3′ reverse primer 5′-CCACACAGCAAAGCAGAAACTCAC-3′ and TaKaRa ExTaq polymerase.

To analyze the deletion mutation, exon5 and 8 of the PTEN gene was amplified using the following PCR forward primers: exon5, 5′-CTCTGGAATCCAGTGTTTCTTT-3′ exon8, 5′-GCAACAGATAACTCAGATTGCC-3′ reverse primer: exon5, 5′- CCAATAAATTCTCAGATCCAGG-3′ exon8, 5′-GTTCTTCATCAGCTGTACTCCT-3′.

To analyze the deletion mutation, Akt gene was amplified using the following PCR forward primers: 5′-GGGTCTGACGGGTAGAGTGT-3′ reverse primer: 5′-GCGCCACAGAGAAGTTGTT-3′.

### Patient Selection

We selected primary NSCLC harboring EGFR mutations, such as exon 19 delE746-A750 and the exon 21 L858R point mutation from the EGFR mutation status records of the Department of Diagnostic Pathology, Kurume University Hospital, Kurume, Japan. These EGFR mutation status records had been determined by DNA direct sequencing or PNA-LNA PCR clamp assay [27], [28].

### Cytological Samples from Cancer Patients

Cell samples were obtained from pleural effusion (5 cases), lymph node fine needle aspiration cytology (2 cases), pericardial effusion (1 case), and cerebrospinal fluid (3 cases), according to a previous study [28]. The pleural effusion and cerebrospinal fluid were centrifuged at 1,500 rpm for 10 min, and the supernatant fluid was removed. The sediment was smeared onto glass slides, and was fixed in 95% ethanol overnight. Fine needle aspiration cytology of lymph nodes was performed using a 23-gauge disposable needle attached to a 10 ml plastic syringe, and the slide was fixed overnight in 95% ethanol.

### Immunostaining for Activating EGFR Mutations

Immunostaining analysis was performed by using anti-EGFR delE746-A750 specific (6B6), the EGFR L858R Mutant-specific (43B2), and total EGFR (D38B1) antibodies (Cell Signaling Technology) as described previously [27].

### Ethics Statement

The study of clinical samples was approved by The Ethical Committee of Kurume University.

**Figure 4:**
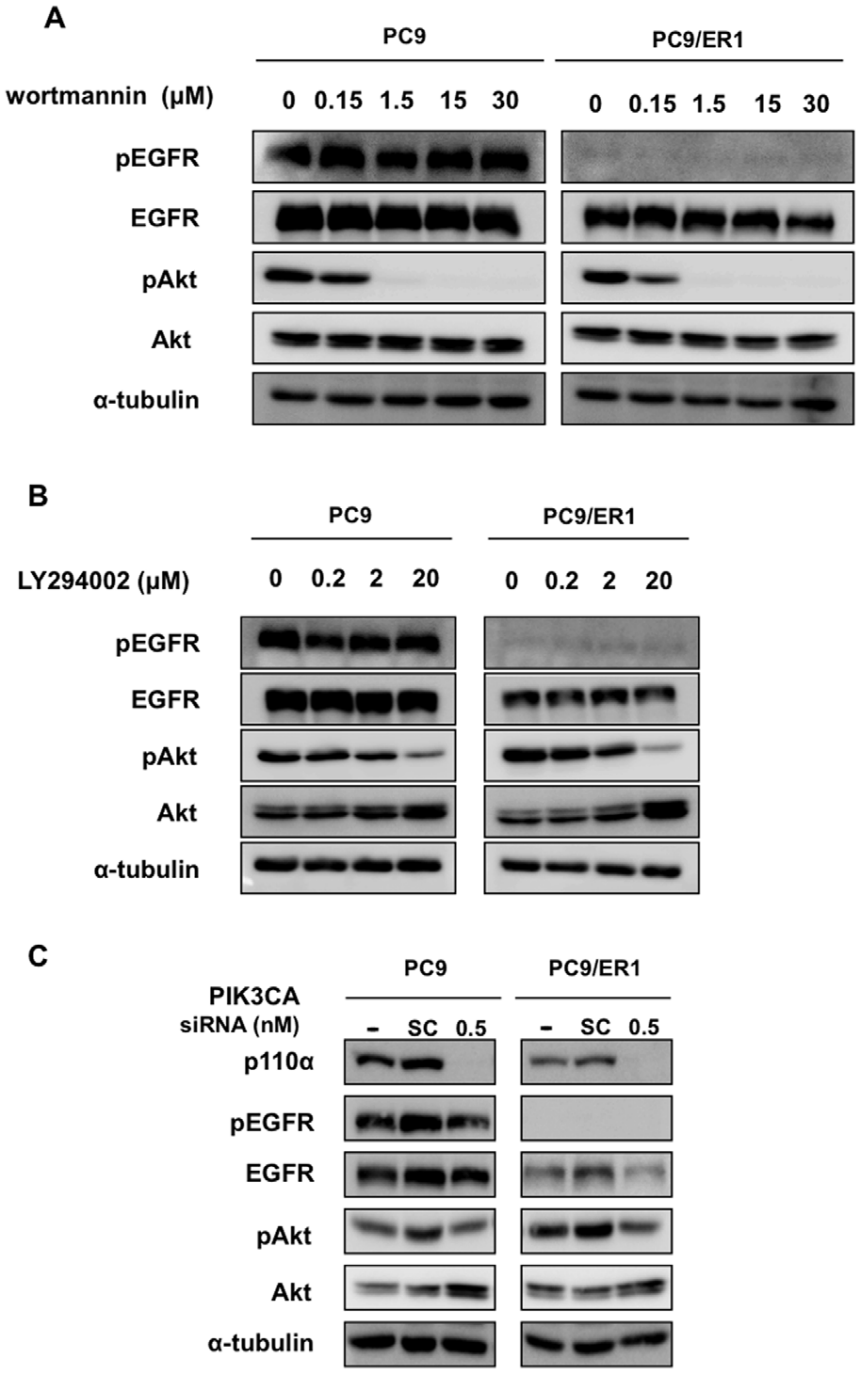
The effect of PI3K inhibitors and PIK3CA knockdown on PC9 and PC9/ER1 cells. A, B, Exponentially growing PC9 and PC9/ER1 cells were exposed to various doses of wortmannin (A) and LY294002 (B) for 5 hr, and followed by Western blot analysis. C. PC9 and PC9/ER1 cells were treated with PIK3CA siRNA or scramble (SC) siRNA for 48 h, and followed by western blot analysis.

## Results

### Establishment of Erlotinib- and Gefitinib-resistant Cell Lines from PC9 and 11–18 Cells

To isolate erlotinib-resistant cell lines from PC9 cells harboring delE746-A750, and from 11–18 cells harboring L858R, both cell lines were cultured in stepwise increasing doses of erlotinib from 0.05 to 10 µM, for approximately 6 months, as described previously [21]. Then, cells were independently selected from each erlotinib-resistant cell line from each plastic dish, to clonally expand one erlotinib-resistant cell line, PC9/ER1, from PC9 cells, and two erlotinib-resistant cell lines, 11–18/ER1-7 and 11–18/ER2-1, from 11–18 cells, respectively. Furthermore, gefitinib-resistant cell lines were also independently isolated and clonally expanded (11–18/GEF10-1 and 11–18/GEF20-1) from 11–18 cells. Dose response curves of drug-resistant cell lines and their parental counterpart to erlotinib or gefitinib showed acquisition of resistance to these drugs in various resistant sublines (Figure S1).

**Figure 5:**
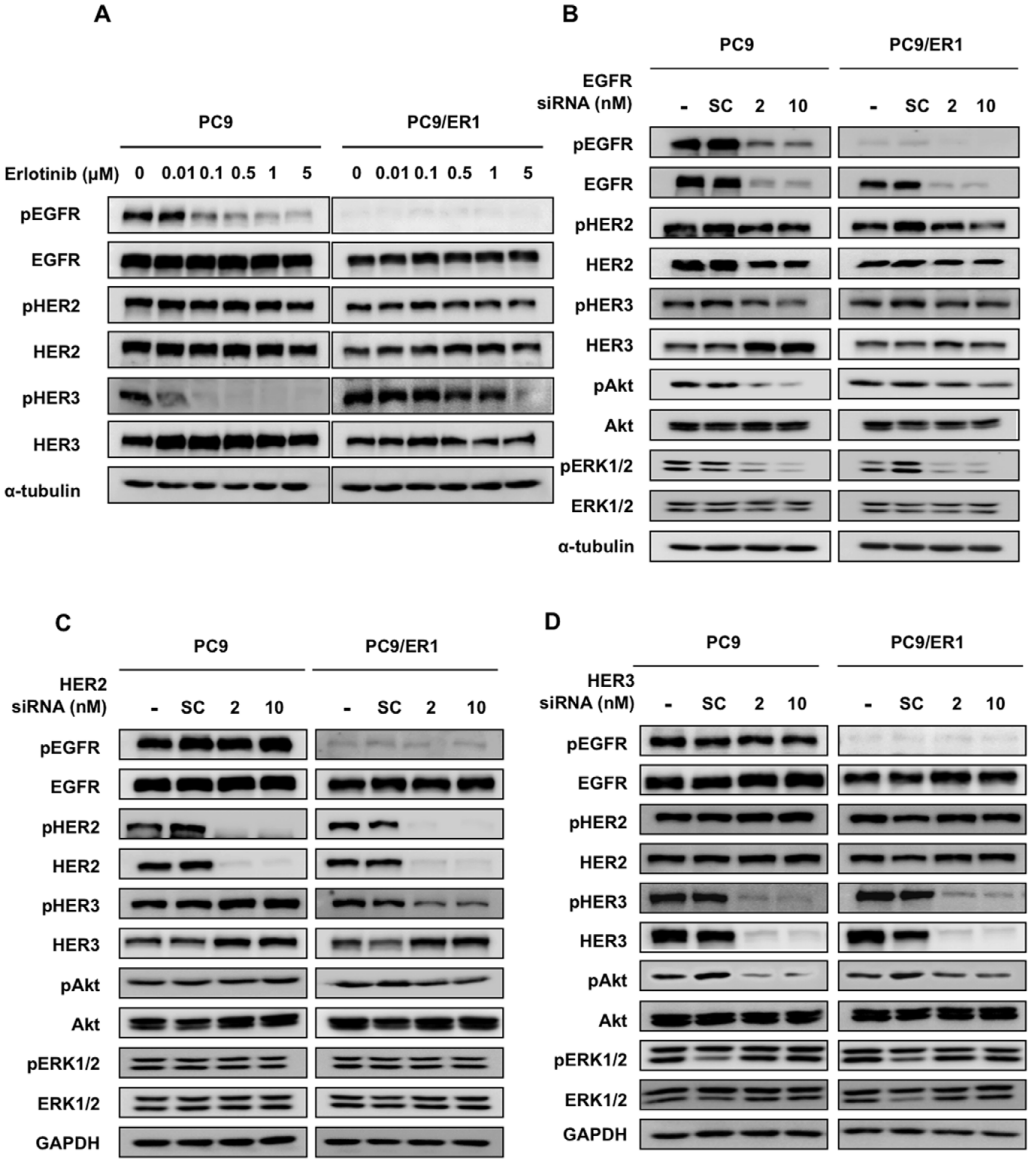
The effect of erlotinib or knockdown of EGFR, HER2, or HER3 by their siRNAs on PC9/ER1 cells. A, Exponentially growing PC9 and PC9/ER1 cells were exposed to various doses of erlotinib for 5 h, and followed by western blot analysis. B, C, D, PC9 and PC9/ER1 cells were treated for 48 hr with 10 nM scramble (sc) siRNA, 2 nM or 10 nM EGFR siRNA, HER2 siRNA or HER3 siRNA respectively, and followed by Western blot analysis.

**Figure 6:**
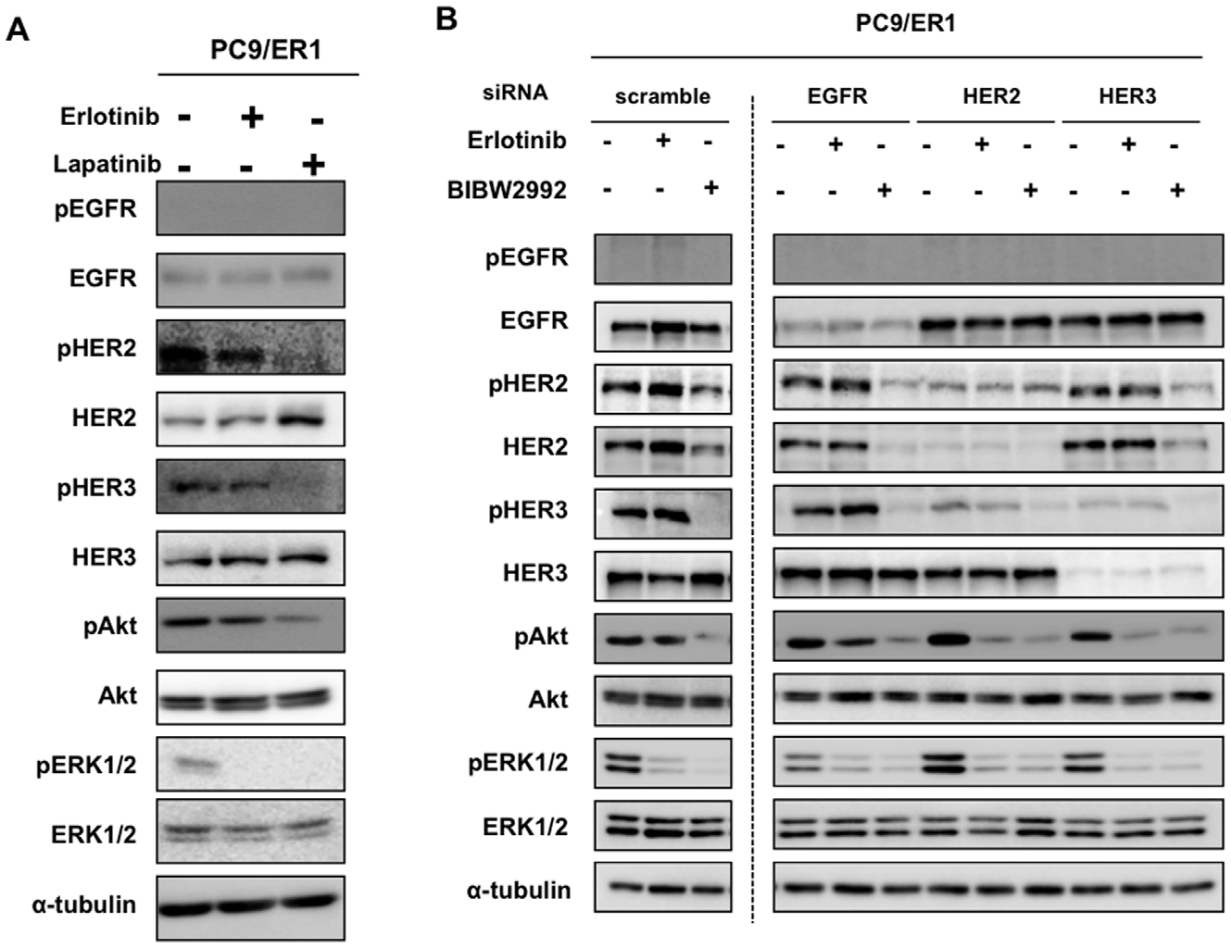
The effect of erlotinib, lapatinib and BIBW2992 on phosphorylation of Akt and EGFR family proteins in PC9/ER1 cells. A, PC9/ER1 cells were treated with or without 1 µM erlotinib, and 5 µM lapatinib for 5 hrs, and followed Western blot analysis. B, PC9/ER1 cells were treated with 10 nM of siRNAs of scrumble and EGFR family genes, and exposed to erlotinib (1 µM) or BIBW2992 (1 µM) for 5 hrs, and followed Western blot analysis.

**Figure 7:**
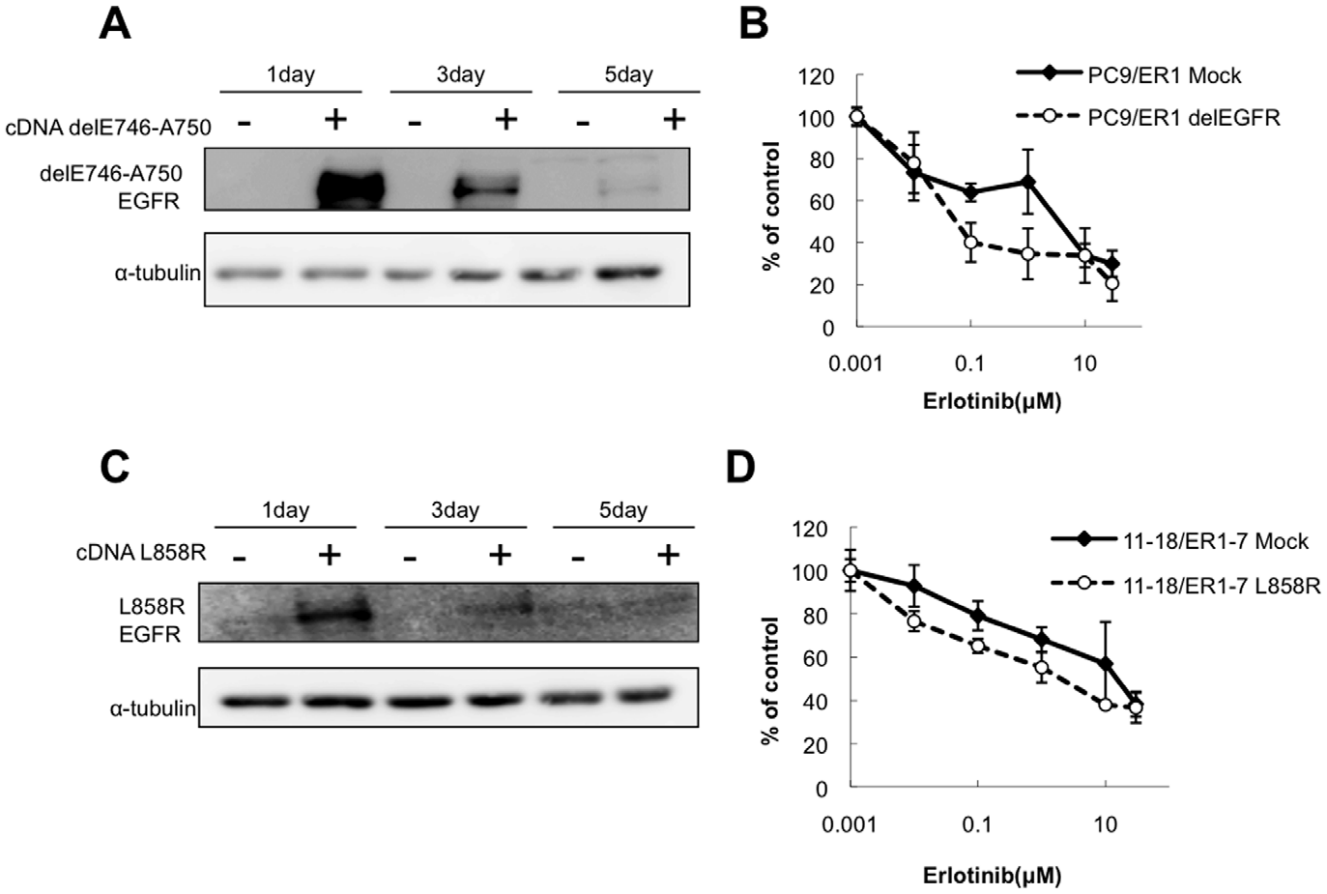
Restoration of sensitivity to erlotinib by expression of activating mutant EGFR in PC9/ER1 and 11–18/ER1-7. A, PC9/ER1 cells were transfected with del EGFR (E746-A750) cDNA, and followed incubation for various days. Western blot analysis was performed with antibodies recognizing del EGFR and total EGFR. B, Dose response curves of mock- and activated mutant EGFR cDNA transfectants of PC9/ER1 were determined by cytotoxicity assay. Each value is average of triplicate dishes ± SD. C, 11–18/ER1-7 cells were transfected with L858R EGFR cDNA, and followed by Western blot analysis with a specific antibody of L858R EGFR. D, Dose response curves of mock- and activated mutant EGFR cDNA transfectants of 11–18/ER1-7. Each values is average of triplicate dishes ± SD.

PC-9/ER1 cells showed 160–250 fold higher resistance to erlotinib and gefitinib, 5 fold higher resistance to lapatinib at most, and about 2,000 fold higher resistance to BIBW2992 (Table 1). 11–18/ER1-7, 11–18/ER2-1, 11–18/GEF10-1, and 11–18/GEF20-1 cells showed 20–110 fold higher resistance to erlotinib and gefitinib and 7 fold higher resistance to lapatinib and BIBW2992 at most (Table 1). On the other hand, all of these resistant cells showed similar sensitivities to SU11274 and cisplatin as their parental counterparts (Table 1).

### Expression of Growth Factor Receptors and their Activating Status in Erlotinib-resistant Cell Lines Established from PC9 and 11–18 Cells

Western blot analysis showed the most striking difference in phosphorylation of EGFR without marked change in phosphorylation status of HER3, c-Met, Akt and ERK1/2 between PC9 and PC9/ER1 cells. On the other hand, relatively lower phosphorylation of EGFR was seen in 11–18/ER1-7 and 11–18/ER2-1 cells than 11–18 cells (Figure 1A).

We next compared activation status of multiple receptor tyrosine kinases including c-Met, Axl, PDGFR and IGF1R which were overexpressed in tumors with EGFR mutations between erlotinib-resistant sublines and their counterparts by using phospho receptor tyrosine kinase (RTK) array [29]. However, there was no difference in activation status of these growth factor receptors including c-Met between drug sensitive and resistant cell lines (Figure S2).

**Figure 8:**
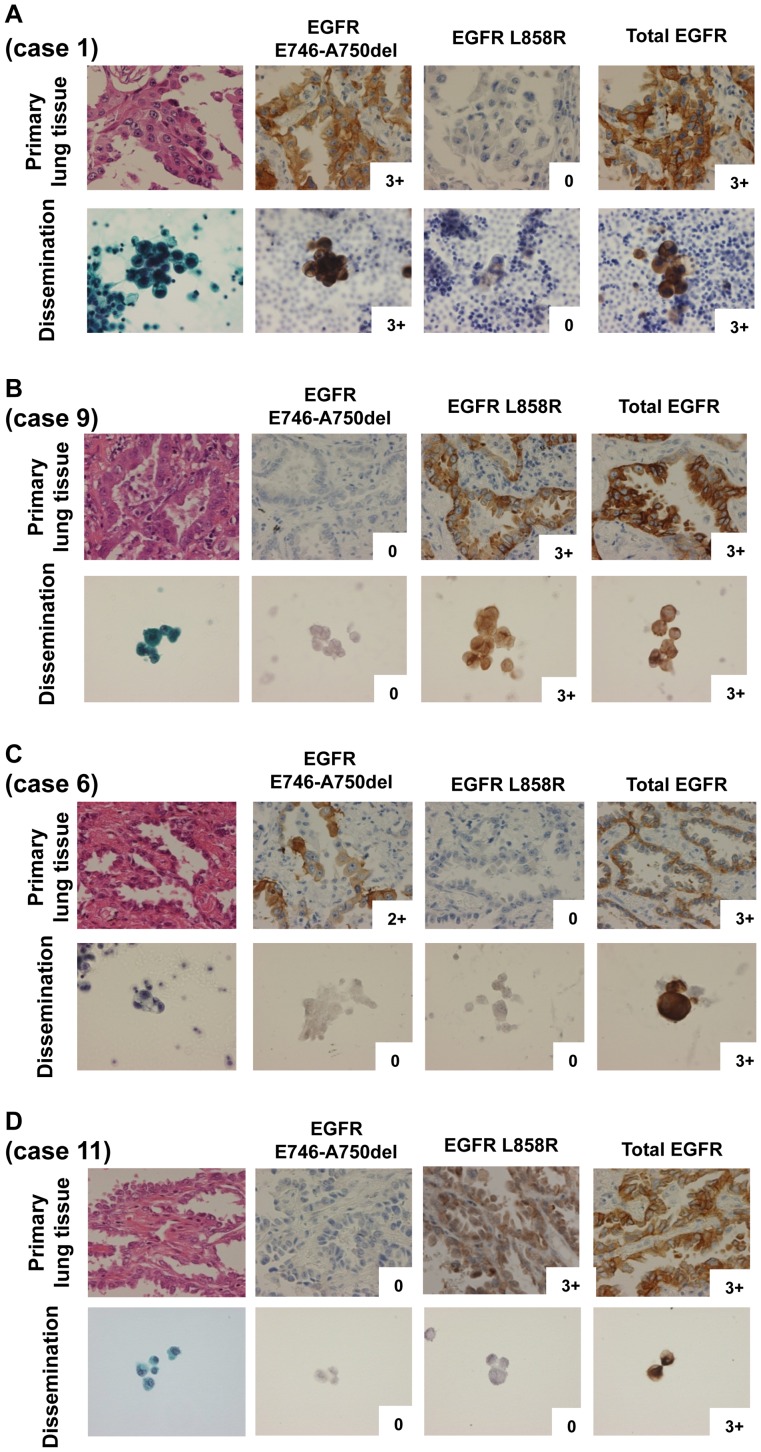
Immunostaining images for EGFR expression in both histological and cytological samples in human NSCLC. Total EGFR antibody stained all four histological and cytological samples. Anti-delE746-A750 antibody stained only cancer cells with the delE746-A750 mutation (A, case 1) (see Table 2), and the EGFR L858R antibody stained only cancer cells with the L858R mutations (B, case 9) in both histological and cytological samples. No staining was evident by both EGFR delE746-A750 and EGFR L858R antibodies in cancer cells without the activating EGFR mutations in cytological samples (C: case 6 and D: case 11).

**Table 2:** Summary of EGFR mutation status in cell samples of refractory cancer patientsa).

Case No.	Cell sample	Primary lung cancer	Refractory cancer		EGFR statusb)		
		EGFR mutation	EGFR mutation	T790M	del	L858R	total EGFR
1	Pleural effusion	del	del	−	3+/3+	0/0	3+/3+
2	Lymph node FNA cytology	del	del	+	3+/3+	0/0	3+/3+
3	Pleural effusion	del	del	+	2+/3+	0/0	3+/3+
4	Pleural effusion	del	del	−	nd/3+	nd/0	nd/3+
5	Celebrospinal fluid	del	del	−	3+/nd	0/nd	3+/nd
6	Lymph node FNA cytology	del	WT	+	2+/0	0/0	3+/3+
7	Pericardial effusion	del	del	−	2+/2+	0/nd	2+/3+
8	Pleural effusion	del	WT	−	3+/0	0/0	3+/3+
9	Pleural effusion	L858R	L858R	−	0/0	3+/3+	3+/3+
10	Celebrospinal fluid	L858R	L858R	+	0/nd	3+/3+	3+/nd
11	Celebrospinal fluid	L858R	WT	−	0/0	3+/0	3+/3+

a) EGFR mutation status including wild-type (WT), E746-A750 del (del), L858R and T790M was determined by both IHC and RNA-LNA PCR clamp assays with 11 clinical samples of cancer patients refractory to gefitinib treatment.

b) EGFR mutation status determined by IHC analysis is presented by scoring (0, 1+, 2+, 3+) (Figure 8) of immunostaining intensity in cancer cells in primary tumor and disseminated samples of 11 patients (Primary tumor sample/Disseminated and/or metastatic sample). nd, not determined.

### Akt Phosphorylation is Not Blocked by Erlotinib in Erlotinib-resistant Cell Lines

We next examined the effect of erlotinib on phosphorylation of EGFR, Akt, and ERK1/2 in erlotinib-resistant cell lines and their parental counterparts (Figure 1B and 1C). In PC9 cells, EGFR, Akt, and ERK1/2 phosphorylation were all inhibited in a dose-dependent manner by erlotinib. However there was almost no inhibition of Akt phosphorylation in PC9/ER1 cells by erlotinib, but ERK1/2 phosphorylation was similarly inhibited as in PC9 cells (Figure 1B). On the other hand, EGFR phosphorylation was found to be equivalently suppressed in 11–18, 11–18/ER1-7, and 11–18/ER2-1 cells by erlotinib. However, as compared with 11–18 cells, Akt phosphorylation in 11–18/ER1-7 and 11–18/ER2-1 cells was not inhibited by erlotinib. By contrast, ERK1/2 phosphorylation was highly sensitive to erlotinib in all 11–18, 11–18/ER1-7, and 11–18/ER2-1 cells (Figure 1C). Acquisition of erlotinib-resistance thus confers constitutive PI3K/Akt phosphorylation in resistant cells from PC9 and 11–18 cells.

### Complete Loss of Activating Mutant EGFR (delE746-A750) Gene in Erlotinib-resistant PC9/ER1 Cells

We then next examined EGFR status in PC9/ER1 cells. Western blot analysis using anti-delE746-A750, L858R, and total EGFR antibodies showed complete loss of mutant EGFR protein expression in PC9/ER1 cells (Figure 2A). Then, the gene profile of wild-type and mutant EGFR between PC9 and PC9/ER1 cells was compared. The direct sequence analysis of exon 19 of the EGFR gene revealed complete loss of only the mutant sequence in PC9/ER1 cells (data not shown). Next, PCR analysis was performed in exon 19 of the EGFR gene by using wild-type and mutation specific primers. PC9 cells contained both wild-type and deletion mutation sequences, indicating heterozygous alleles for wild-type and mutant EGFR, while there was only a wild-type sequence in PC9/ER1 cells (Figure 2B). Exon 19 of the EGFR gene was further amplified, and the analysis of these DNA samples in the gel consistently showed the presence of only the wild-type sequence in exon 19 of the EGFR gene in PC9/ER1 cells, although PC9 cells contained both the deletion and wild-type sequence (Figure 2C). Taken together, the PC9/ER1 cells showed complete loss of the mutant EGFR gene by acquisition of drug resistance to erlotinib.

**Figure 9:**
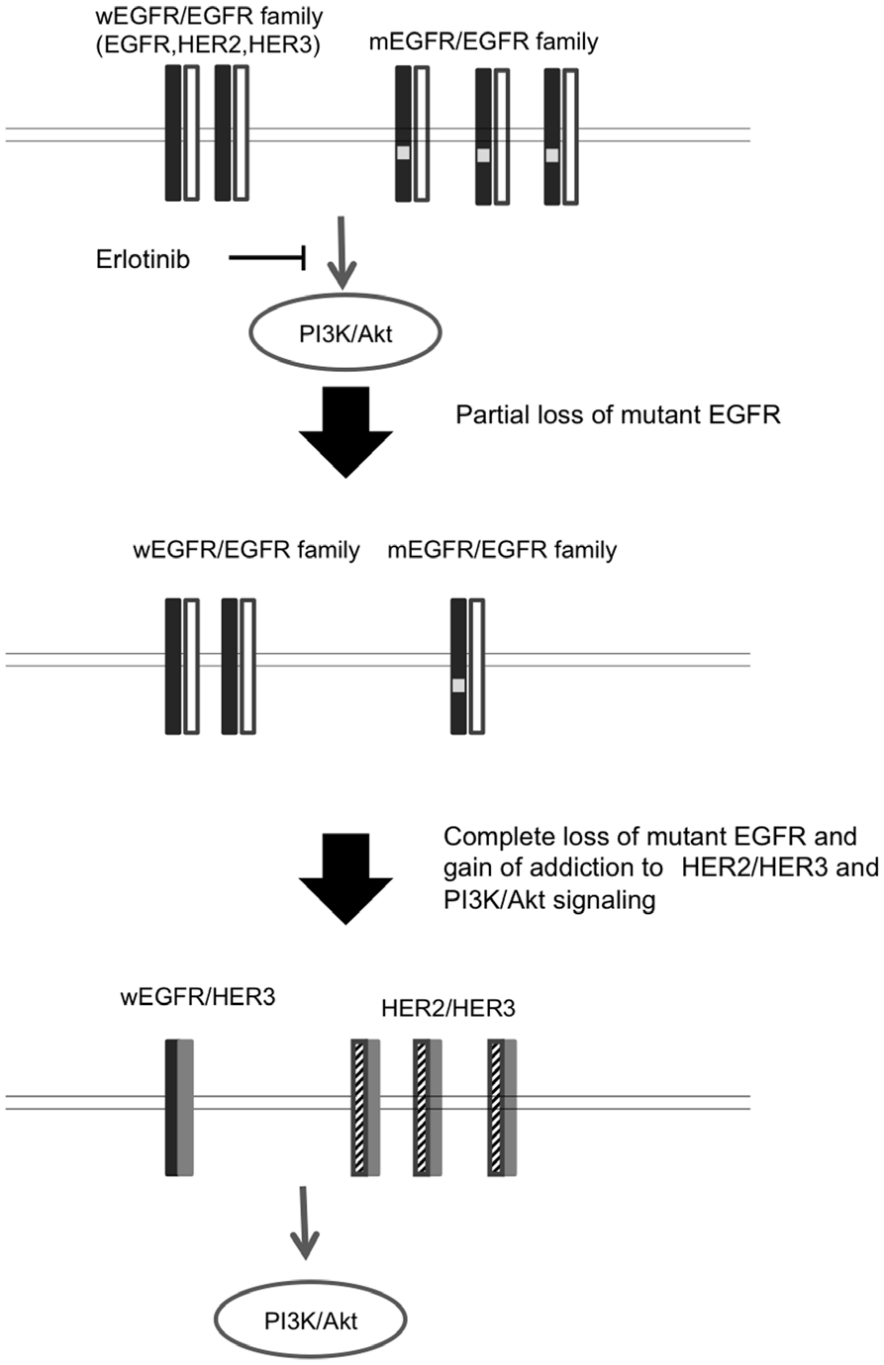
Our hypothetic model how drug resistance to erlotinib is acquired in lung cancer cells harboring activated mutant EGFR (mEGFR). Cell proliferation and survival of human lung cancer cells harboring activated mutant EGFR (PC9 and 11–18 cells) closely depend upon EGFR-driven PI3K/Akt pathway, and this proliferation/survival is highly susceptible to erlotinib and other EGFR TKIs. First, there is partial or complete loss of mEGFR gene allele in drug-resistant cell lines, and then gain of addiction to HER2/HER3 and PI3K/Akt signaling (PC9/ER1 cells). However, more definitive analysis on resistant cell lines of 11–18 is required because 11–18 resistant cell lines show only partial loss of mEGFR.

### Partial Loss of the Activating Mutant EGFR (L858R) Gene in Erlotinib- or Gefitinib-resistant Cell Lines from 11–18

We further compared expression levels of wild-type EGFR and mutant EGFR by a specific antibody that recognizes the L858R mutant EGFR by western blot analysis. Compared with the parental 11–18 cells, expression of the mutant L858R EGFR protein was relatively lower versus total cellular EGFR levels (Figure 3A).

We next examined whether activating mutant EGFR gene in 11–18/ER1-7 and 11–18/ER2-1 cells was affected by the acquisition of erlotinib resistance or not. DNA sequence analysis showed the presence of the mutation (L858R) both in the parental and resistant cells (Figure 3B, arrows indicate nucleotide 2573), although alternation of the peak heights on nucleotide 2573 was obvious. Altered ratio of wild-type to mutant EGFR gene was also observed by PLACE-SSCP analysis [23], [24], as exemplified in Figure 3C. This assay showed two independent peaks, one for wild-type and another for mutant EGFR gene, both in 11–18 and erlotinib-resistant cells. However, the peak height ratio (Rh) of the two resistant cell lines was clearly different. By adopting mixing strategy, that is, mixing the DNAs of HUVECs carrying 2 copies of wild-type EGFR gene with that of resistant cells, the change in copy number of the allele could be quantified as described in Materials and Methods. The results indicated about a 50% decrease of the mutant EGFR gene without apparent change of the wild-type EGFR gene copy (Figure 3C).

We also examined whether selection by drug resistance to gefitinib also induced similar changes of decreased expression of the activating EGFR gene. Two gefitinib-resistant cell lines, 11–18/GEF10-1 and 11–18/GEF20-1, showed increased EGFR protein expression with relatively decreased expression of HER2 and pHER2 in comparison with their parental 11–18 cells (Figure S3A). As compared with the parental 11–18 cells, Akt phosphorylation in 11–18/GEF10-1 and 11–18/GEF20-1 was not affected by gefitinib when phosphorylation of EGFR and ERK1/2 was similarly inhibited by gefitinib (Figure S3B). Western blot analysis with the anti-L858R antibody showed decreased expression of the mutant EGFR and similar expression of the total EGFR in two resistant cell lines as compared with 11–18 cells (Figure S3C). Next, we performed DNA sequence analysis and found an alternating peak height on nucleotide 2573 in gefitinib-resistant cells (Figure S3D). PLACE-SSCP analysis also revealed a decreased mutant EGFR gene copy without apparent changes in wild-type EGFR gene copy, and quantitative analysis indicating about a 50% decrease of the mutant EGFR gene in gefitinib-resistant cells (Figure S3E). From these analyses of erlotinib- or gefitinib-resistant cells lines, acquisition of drug resistance may be mediated through a decreased mutant EGFR gene copy.

### Knockdown of HER2 or HER3 Sensitizes the Constitutive Activation of Akt to Erlotinib in PC9/ER1 Cells

There was almost complete loss of mutant EGFR gene in PC9/ER1 whereas there was only partial loss of the mutant EGFR gene in erlotinib-resistant cell lines derived from 11–18. We further analysed more in detail any mechanism underlying acquirement of erlotinib resistance in PC9/ER1. We examined the effect of PI3K inhibitors, wortmannin and LY294002 on Akt activation in PC9 and PC9/ER1 cells (Figure 4A and 4B). Both PI3K inhibitors similarly inhibited phosphorylation of Akt, indicating that activated Akt is similarly susceptible to both inhibitors in PC9/ER1 and PC9 cells. We also confirmed specific suppression of Akt activation in both PC9 and PC9/ER1 cells when treated with PIK3CA siRNA (Figure 4C). Furthermore, sequence analysis revealed that there was no mutation in hot spots of PIK3CA, PTEN and Akt gene (data not shown). The constitutive Akt activation in PC9/ER1 seems not to be due to altered PI3K/Akt pathway itself.

We finally examined which molecules among EGFR, HER2 or HER3 could be responsible for the constitutive Akt activation in erlotinib-resistant PC9/ER1 cells. We found phosphorylation of HER3 was not suppressed by erlotinib in PC9/ER1 compared to PC9 (Figure 5A). We then examined whether knockdown of EGFR, HER2 or HER3 by their cognate siRNAs could modulate activation of Akt and EGFR family proteins. Knockdown of EGFR resulted in markedly decreased activation of Akt only in PC9 cells but not in PC9/ER (Figure 5B). On the other hand, knockdown of HER3 could suppress activation of Akt in both PC9 and PC9/ER (Figure 5D). Furthermore activation of HER3 was markedly suppressed by HER2 knockdown only in PC9/ER (Figure 5B). These results suggest that HER3 together with HER2 signaling are responsible for constitutive activation of PI3K/Akt in acquired resistance to erlotinib in PC9/ER.

We further examined whether lapatinib, a dual kinase inhibitor of EGFR and HER2, could suppress Akt activation in PC9/ER1. Treatment with lapatinib inhibited phosphorylation of Akt and HER3 while erlotinib did not (Figure 6A). We next examined the effect of erlotinib or a pan-tyrosine kinase inhibitor of all EGFR family, BIBW2992 [30], on Akt phosphorylation in PC9/ER1 when each EGFR, HER2 or HER3 was silenced (Figure 6B). The phosphorylation of HER2, HER3 and Akt was all suppressed by BIBW2992 alone. On the other hand, the phosphorylation of Akt was inhibited by erlotinib with either HER2 or HER3 knockdown. Furthermore, HER2 knockdown resulted in a marked inhibition of HER3 phosphorylation, suggesting that PC9/ER1 cells gain addiction to HER2/HER3 signaling (Figure 6B).

We finally examined whether expression of activating mutant EGFR could restore drug sensitivity to erlotinib in drug resistant cell lines, PC9/ER1 and 11–18/ER1-7. Transient transfection of del (E746-A750) EGFR cDNA induced enhanced expression of activated mutant EGFR in PC9/ER1 (Figure 7A). Overexpression of del (E746-A750) EGFR cDNA overcame drug resistance to erlotinib in PC9/ER1 (Figure 7B). Furthermore, transfection of another activated mutant L858R EGFR cDNA also induced enhanced expression (Figure 7C) and restored drug sensitivity to erlotinib in 11–18/ER1-7 cells (Figure 7D).

### Loss of Activating Mutant EGFR in Refractory Non-small-cell Lung Cancers

Figure 8 showed representative IHC images for wild-type, delE746-A750, and L858R EGFR expression in primary lung cancer tissues (Figure 8, each upper panel), and also cancer cells in pleural effusion or cerebrospinal fluid in recurrent patients after treatment with gefitinib (Figure 8, each lower panel). As shown in Table 2, out of 11 patients who first received gefitinib after lung surgery and then showed recurrence, 8 patients had the delE746-A750 mutation and 3 had L858R mutation in their primary lung tumors. Four had the T790M mutation in dissemination or metastatic cytological samples. Out of 11 refractory patients, 2 of the 8 cases that had harbored the delE746-A750 showed loss of the activating EGFR mutation, and 1 of the 3 cases that had harbored L858R showed loss of the activating mutation (Table 2). In one case (case 6), both T790M mutation and wild-type EGFR expression were observed. There was no disagreement between the expression of EGFR mutation-specific antibodies and detection of EGFR mutations by sequence analysis using PNA-LNA PCR clamp assay in all samples tested in this study.

## Discussion

Activating EGFR mutations, such as delE746-A750 and L858R, cause lung cancer cells closely couple EGFR with cell proliferation or survival [5], [6], [15]. The presence of activating EGFR mutations is closely associated with a more favorable outcome following treatment with EGFR-targeted drugs [15]. In our present study, erlotinib-resistant cell lines were established; PC9/ER1 from PC9 cells harboring delE746-A750 mutation, and 11–18/ER1-7 and 11–18/ER2-1 from 11–18 cells harboring L858R mutation. Gefitinib-resistant cell lines were also established (11–18/GEF10-1 and 11–18/GEF20-1) from 11–18 cells.

Gene amplification and increased copy number of the EGFR gene associated with the response rate to EGFR-targeted drugs in NSCLC, breast cancer and colon cancer [31], [32]. However, in these studies, specific gene copy of the wild-type and mutant EGFR gene allele was not independently determined. By using allele-specific PCR analysis and PLACE-SSCP analysis, we found that erlotinib- or gefitinib-resistant cell lines showed either complete or partial loss of activating mutant EGFR gene allele versus wild-type of EGFR gene allele, accompanying by constitutive activation of PI3K/Akt less susceptible to effect of erlotinib or gefitinib. Erlotinib-resistant cell line (PC9/ER1) showed almost complete loss of mutant EGFR gene allele, but drug resistant cell lines from 11–18 showed partial loss of mutant EGFR gene allele.

In this study, we have further analysed the underlying mechanism for drug resistance in PC9 cells, and compared with drug resistance relevant characteristics of resistant cell lines of 11–18. An erlotinib-resistant cell line (PC9/ER1) showed complete loss of mutant EGFR gene allele, and harbored only wild-type EGFR (Figure 2). The loss of activating mutant EGFR is followed by constitutive activation of its downstream PI3K/Akt signaling pathway that is not inhibited by erlotinib. The PI3K/Akt activation independent of activating mutant EGFR therefore seems to play essential role in acquisition of drug resistance to EGFR-targeted drugs in PC9/ER1 cells. Forced expression of activated mutant (delE746-A750) EGFR cDNA restored sensitivity to erlotinib in PC9/ER1 cells, supporting the initial discovery that activating mutant EGFR gene plays a key role in drug sensitivity to gefitinib [5], [6], [7]. Furthemore, in erlotinib- or gefitinib-resistant cell lines of 11–18, PLACE-SSCP analysis demonstrated apparent decrease of more than 50% of the mutant EGFR gene copy, together with relatively decreased levels of the mutant EGFR protein, as compared with their parental cell line. Transfection of activating mutant EGFR cDNA into erlotinib-resistant subline of 11–18 also restored sensitivity to erlotinib, suggesting again the close connection of the partial loss of mutant EGFR gene with acquisition of drug resistance in 11–18.

One could argue why the loss of activating mutant EGFR gene allele confer drug-resistant phenotype and PI3K/Akt activation. Acquired drug resistance to kinase inhibitors in general can lead to reactivation of the target protein, activation of up-stream or down-stream effectors, and/or activation of bypass pathway [33]. Of these pleiotropic proteins involving acquired resistance to EGFR-targeted drugs, we examined whether other EGFR family proteins could play a role in constitutive activation of PI3K/Akt during acquirement of erlotinib resistance. Of three EGFR family proteins, phosphorylation EGFR and HER3 was susceptible to the inhibitory effect of erlotinib in PC9, but phosphorylation of HER3 was not inhibited to erlotinib in its drug-resistant counterpart (Figure 5A). In the parental PC9 cells, knockdown of either EGFR or HER3 resulted in decreased expression of pAkt (Figure 5B and 5C), consistent with the notion that activated EGFR mutation in association with HER3 or HER2 highly sensitize the Akt phosphorylation to EGFR-targeted drugs [15], [16]. HER2 knockdown itself however did not affect phosphorylation of Akt in PC9 cells. In PC9/ER1 cells, knockdown of HER2 suppressed expression of pHER3 and pAkt while knockdown of EGFR, mostly wild-type EGFR, suppressed expression of pHER2 and pAkt, and only slightly that of pHER3 (Figure 5B and 5C). Furthemore, knockdown of HER3 suppressed phosphorylation of Akt in PC9/ER1 cells (Figure 5D). On the other hand, treatment with lapatinib, a dual kinase inhibitor, or BIBW2992, a pan-kinase inhibitor, suppressed phosphorylation of HER2, HER3 and Akt in PC9/ER1 cells (Figure 6A and 6B). Figure 6B shows that phosphorylation of Akt is highly susceptible to erlotinib when HER2 or HER3 was silenced in PC9/ER1 cells. By contrast, phosphorylation of Akt was partially suppressed by erlotinib in EGFR-knockdowned PC9/ER1cells (Figure 6B).

During selection of drug resistant cell lines from PC9, HER3 and HER2 thus seem to activate PI3K/Akt pathway in erlotinib-resistant cells, and this HER2/HER3-driven Akt activation pathway may play a pivotal role in acquired resistance to erlotinib in PC9/ER1 cells. HER3 and HER2 in its close connection with wild-type EGFR may also in part involve acquirement of drug resistance (Figure 9). A relevant study has previously demonstrated that HER2/HER3-driven signaling pathway limits sensitivity to EGFR targeted drugs in cancer cells [34]. On the other hand, exogenous transfection of activated mutant EGFR cDNA partially restored drug sensitivity to erlotinib in 11–18/ER1-7 cells and knockdown of HER3 or HER2 also sensitized cells to erlotinib by inhibiting phosphorylation of Akt. Similar mechanism as in PC9 might be involved in acquirement of drug resistance to erlotinib in 11–18. However, more precise study should be further required to understand the underlying mechanism for drug resistance in 11–18.

During acquirement of drug resistance to EGFR-targeted drugs, activation by bypass mechanisms and genomic alternation affecting up-stream or down-stream effectors are also involved [33]. In addition to PI3K/Akt activation independent of activated mutant EGFR in erlotinib- and/or gefitinib-resistant cell lines, we also examined whether other mechanisms could play any role in acquirement of drug resistance. Alternative activation of c-Met and IGF1R abrogate the close association of EGFR with cell survival, accompanied by tumor growth that is independent of EGFR [16], [19], [20], [35]. In particular, overexpression of IGF1R has been in EGFR-TKI resistant cell lines derived from 11–18 [36]. Our erlotinib- and gefitnib-resistant cell lines show similar sensitivity to c-Met-TKI (SU11274) (Table 1), and the IGF1R-TKI (Picropodophyllin) (unpublished data), as their parental cell lines. Moreover, from RTK array, activation status of IGF1R, AXL, c-Met, and PDGFR was not stimulated in resistant cells lines as compared with their parental counterpart (Figure S2), suggesting that these kinase pathways are not likely involved. Furthermore, DNA sequence analysis showed no acquisition of a representative secondary mutation of drug resistance in lung cancer cells, T790M mutation. Phosphorylation of Akt was found to be susceptible to PIK3CA knockdown, and also PI3K inhibitors, wortmannin and LY294002 in PC9/ER1 (Figure 4) [37]–[39]. In addition, neither activating mutation in PIK3CA nor PTEN mutation was observed. It seems likely that PI3K/Akt pathway is not mutated during selection of drug resistant cell lines.

Eleven NSCLC patients with adenocarcinomas harbored activating EGFR mutations, including E746-A750del and L858R, and became refractory to treatment with gefitinib (Table 2). In these patients, pleural dissemination of cancer cells was observed in the pleural cavity and cerebrospinal fluid after gefitinib treatment. Out of 11patients, 3 cases showed loss of activating mutant EGFR after recurrence. However, 1 out of 3 cases harbored wild-type EGFR with T790M mutation (case 6). The loss of activating mutant EGFR gene without affecting on the wild-type EGFR gene copy might be responsible for acquisition of drug resistance to EGFR-TKIs in NSCLC patients. However, this is highly speculative because there is no genomic analysis of wild-type and mutant EGFR gene copy in these clinical samples. Furthermore, this frequency for the loss of the mutant EGFR in recurrent NSCLC patients might be overestimated because the number of cancer cells in pleural and cerebrospinal fluids tested by cytological analysis was limited. Further study should be required to confirm whether such loss of mutant EGFR gene copy is specifically responsible for acquirement of drug resistance in patients with lung cancer.

In conclusion, we observed the loss of the mutant EGFR gene allele accompanying by constitutive Akt activation in the presence of erlotinib during the selection of drug resistant cell lines. Our present study may propose a novel mechanism for acquisition of drug resistance to erlotinib or gefitinib in lung cancer. Decreasing gene copy of the activating mutant EGFR may induce dysregulation of the close coupling of EGFR with cell survival signaling. Our study indicates that the alternative activation of HER3/HER2 is responsible for acquisition of drug resistance (Figure 9). Further analysis is important to evaluate how the above mechanism for the altered gene copy number of wild-type or mutant EGFR gene could be induced during acquisition of drug resistance to EGFR-targeted drugs in lung cancer cells in patients.

## Supporting Information

Figure S1 **Comparison of the sensitivity to erlotinib or gefitinib in these resistant cell lines derived from PC9 or 11–18 cells.** A, B, C, Dose-response curves of PC9 and PC9/ER1 cells (A) and 11–18, 11–18/ER1-7, 11–18/ER2-1 cells (B) to erlotinib, and 11–18, 11–18/GEF10-1 and 11–18/GEF20-1(C) to gefitinib. Sensitivity to erlotinib or gefitinib was determined by WST assay in the presence of various doses of these drugs for 72 hr. Each value is the average of triplicate wells (±SD).
(TIF)

Figure S2 **Detection of the phosphorylation status of 42 RTKs in erlotinib-resistant cell lines and their parental cell lines using human phospho-RTK array.** A, B, PC9 and PC9/ER1 (A) and 11–18, 11–18/ER1-7, 11–18/ER2-1 (B) cell lysate were incubated with membranes containing antibodies to 42 different RTKs. The membranes were washed and incubated with a pan anti-phospho-tyrosine antibody to measure the levels of active receptor.
(TIF)

Figure S3 **Comparison of expression of EGFR family proteins and their down-stream signaling molecules, DNA sequence analysis, and gene copy for wild-type and mutant EGFR gene between gefitinib-resistant cell lines and their parental 11–18 cells.** A, Comparison of the expression of EGFR, p-EGFR, HER2, p-HER2, HER3, p-HER3, PTEN, Akt, p-Akt, ERK1/2, and p-ERK1/2 in 11–18, 11–18/GEF10-1, and 11–18/GEF20-1 cells by western blot analysis. B, Exponentially growing 11–18, 11–18/GEF10-1, and 11–18/GEF20-1 cells were exposed to various doses of erlotinib for 5 hr, and followed by Western blot analysis. C, Western blots showing expression of L858R EGFR protein in 11–18 cells and resistant clones. Expression levels of mutant EGFR (L858R), total EGFR, and L858R versus total EGFR (L858R/total EGFR) are normalized by their expression levels in 11–18 cells. D, Comparison of DNA sequences of 15 bases responsible for the L858R mutation in the EGFR gene exon 21 in 11–18, 11–18/GEF10-1, and 11–18/GEF20-1 cells. E, Comparison of gene copy of wild-type and mutant EGFR between 11–18 cells and gefitinib-resistant counterparts by PLACE-SSCP. Two peaks show wild-type (WT) and mutant (Mut) EGFR gene (a). Copy number of wild-type and mutant EGFR gene is summarized (b).
(TIF)
