# Supplementary figures and images for: Loss of Activating EGFR Mutant Gene Contributes to Acquired Resistance to EGFR Tyrosine Kinase Inhibitors in Lung Cancer Cells
Source: PLoS One. 2012 Jul 17;7(7):e41017. doi: 10.1371/journal.pone.0041017 (PMC3398867; doi:10.1371/journal.pone.0041017)

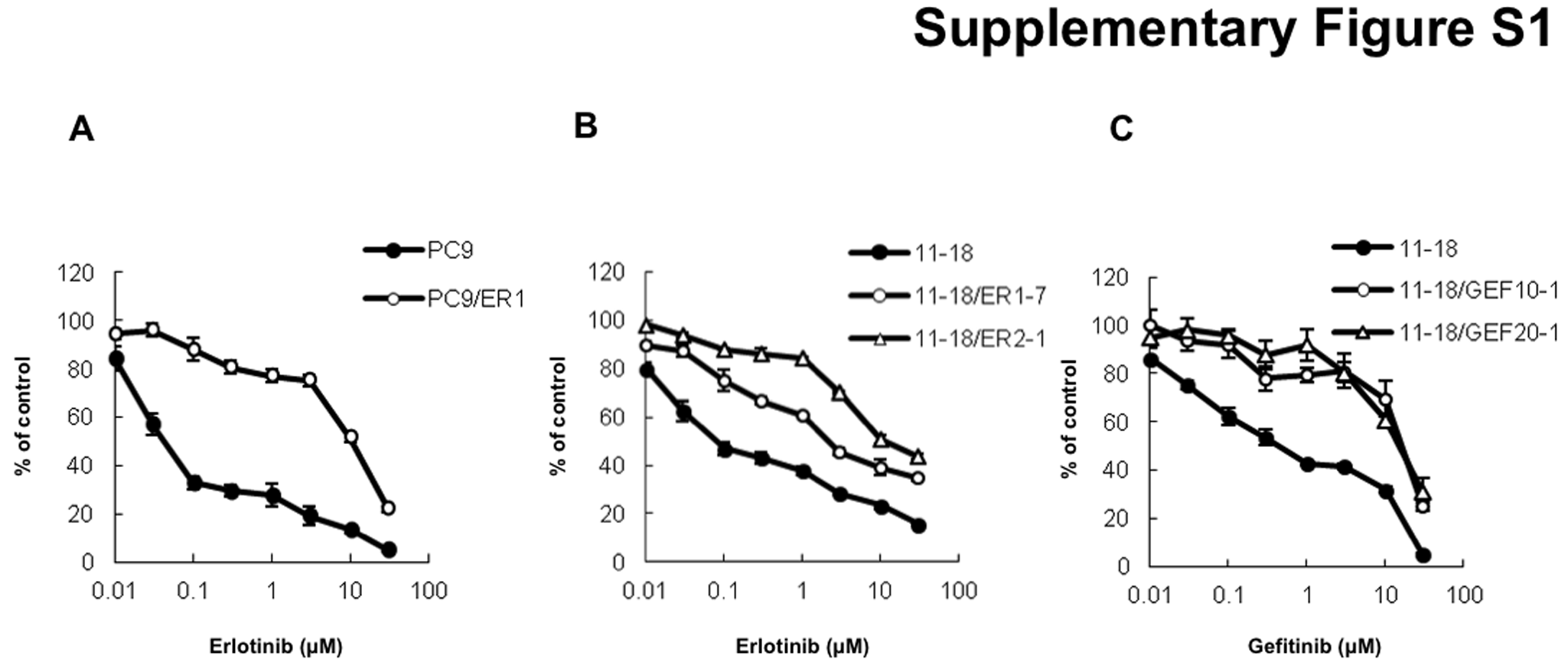

Supplement: Figure S1 — Comparison of the sensitivity to erlotinib or gefitinib in these resistant cell lines derived from PC9 or 11–18 cells. A, B, C, Dose-response curves of PC9 and PC9/ER1 cells (A) and 11–18, 11–18/ER1-7, 11–18/ER2-1 cells (B) to erlotinib, and 11–18, 11–18/GEF10-1 and 11–18/GEF20-1(C) to gefitinib. Sensitivity to erlotinib or gefitinib was determined by WST assay in the presence of various doses of these drugs for 72 hr. Each value is the average of triplicate wells (±SD). (TIF) [file pone.0041017.s001.tif]

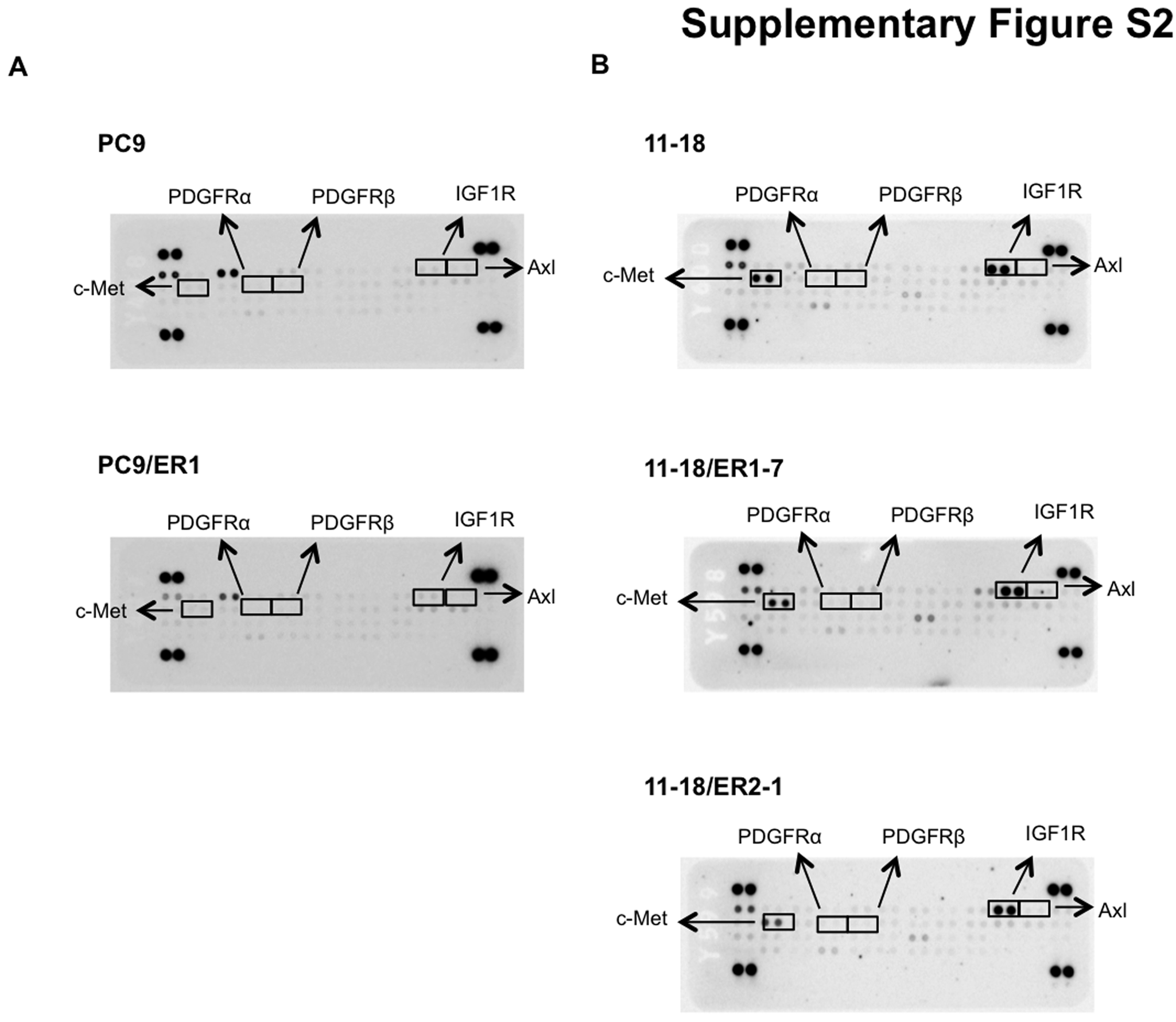

Supplement: Figure S2 — Detection of the phosphorylation status of 42 RTKs in erlotinib-resistant cell lines and their parental cell lines using human phospho-RTK array. A, B, PC9 and PC9/ER1 (A) and 11–18, 11–18/ER1-7, 11–18/ER2-1 (B) cell lysate were incubated with membranes containing antibodies to 42 different RTKs. The membranes were washed and incubated with a pan anti-phospho-tyrosine antibody to measure the levels of active receptor. (TIF) [file pone.0041017.s002.tif]

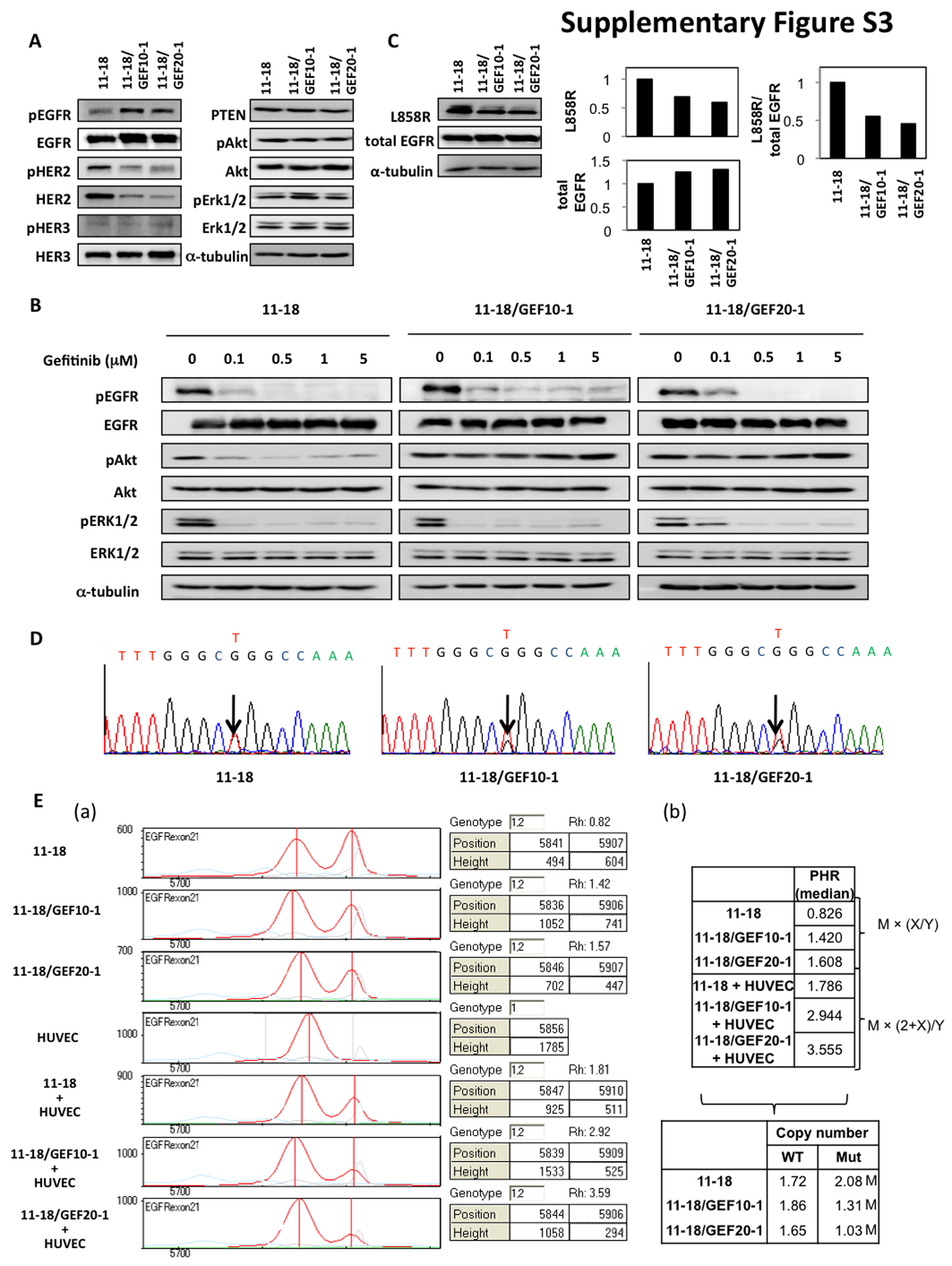

Supplement: Figure S3 — Comparison of expression of EGFR family proteins and their down-stream signaling molecules, DNA sequence analysis, and gene copy for wild-type and mutant EGFR gene between gefitinib-resistant cell lines and their parental 11–18 cells. A, Comparison of the expression of EGFR, p-EGFR, HER2, p-HER2, HER3, p-HER3, PTEN, Akt, p-Akt, ERK1/2, and p-ERK1/2 in 11–18, 11–18/GEF10-1, and 11–18/GEF20-1 cells by western blot analysis. B, Exponentially growing 11–18, 11–18/GEF10-1, and 11–18/GEF20-1 cells were exposed to various doses of erlotinib for 5 hr, and followed by Western blot analysis. C, Western blots showing expression of L858R EGFR protein in 11–18 cells and resistant clones. Expression levels of mutant EGFR (L858R), total EGFR, and L858R versus total EGFR (L858R/total EGFR) are normalized by their expression levels in 11–18 cells. D, Comparison of DNA sequences of 15 bases responsible for the L858R mutation in the EGFR gene exon 21 in 11–18, 11–18/GEF10-1, and 11–18/GEF20-1 cells. E, Comparison of gene copy of wild-type and mutant EGFR between 11–18 cells and gefitinib-resistant counterparts by PLACE-SSCP. Two peaks show wild-type (WT) and mutant (Mut) EGFR gene (a). Copy number of wild-type and mutant EGFR gene is summarized (b). (TIF) [file pone.0041017.s003.tif]
